# Phase 0 trials/ Intra-Target-Microdosing (ITM) and the lung: a review

**DOI:** 10.1186/s12890-024-03193-5

**Published:** 2024-08-29

**Authors:** Tom M. Quinn, Annya M. Bruce, Tal Burt, Kevin Dhaliwal

**Affiliations:** 1 Baillie Gifford Pandemic Science Hub, Centre for Inflammation Research, Institute for Regeneration & Repair, Edinburgh BioQuarter, University of Edinburgh, Edinburgh, UK; 2 Department of Respiratory Medicine, Western General Hospital, Edinburgh, UK; 3 Burt Consultancy, LLC, New York, NY USA; 4 Department of Respiratory Medicine, New Royal Infirmary of Edinburgh, Edinburgh BioQuarter, Edinburgh, UK

**Keywords:** Respiratory Medicine, Drug development, Experimental medicine, Phase 0/ microdosing

## Abstract

The COVID-19 pandemic has highlighted the importance of efficient drug discovery in respiratory disease. The traditional set up of clinical trials is expensive and allows for significant attrition of new drugs, many of which undergo extensive safety testing before being abandoned for lack of efficacy. Phase 0 trials, named as they sit between pre-clinical research and phase I, allow for the testing of sub-clinical microdoses in humans to gather early pharmacokinetic (PK), pharmacodynamic (PD) and mechanistic data, before deciding on which drugs to advance further. This early data can improve the efficiency and cost effectiveness of drug development and reduce the extent of animal testing. Phase 0 trials traditionally have utilised sub-therapeutic microdoses of compounds administered intravenously with readouts focusing on PK - measured using highly sensitive methods such as accelerator mass spectrometry (AMS) and liquid chromatography tandem mass spectrometry (LC-MS/MS) of peripheral blood, as well as whole-body positron emission tomography (PET). Mathematical models allow for extrapolation of this PK data to support the further testing of larger, systemically effective doses. However, this extrapolation method is limited at providing robust PD or target engagement/ mode of action data. Using an Intra-Target Microdosing (ITM) approach, a small compartment of the body (about 1% or less) is exposed to potentially clinically active local concentrations. This allows for the collection of PD data, evidence of target cell engagement, as well as the opportunity to extrapolate systemic PK and PD data. This approach has the potential within the pulmonary system for the study and rapid and cost-effective development of new and repurposed drugs.

## Introduction

Global aging and expanding human populations require more efficient and rapid drug discovery. Increasing numbers of people are living with chronic disease and as the COVID-19 pandemic has highlighted, there is the potential for new pathogens, as well as antimicrobial resistance to established pathogens. The traditional set up of phase I, II and III clinical trials have routinely demonstrated poor cost-effectiveness and allows for significant attrition of ineffective new drugs [1, 2], many of which undergo extensive safety and efficacy testing before being abandoned for lack of efficacy or excessive toxicity. Phase 0 trials, named as they sit between pre-clinical research and phase I, allow for the testing of tiny, sub-clinical (e.g., 1/100^th^ of the estimated minimal clinical dose or less) doses in humans to gather early pharmacokinetic (PK) and pharmacodynamic (PD) data [3], before choosing promising drugs to advance to formal phase I trials. It is especially effective in developmental scenarios where there are multiple pre-clinical candidates that cannot be separated based on the pre-clinical data alone. Phase 0 & I trials can operate in parallel, with compounds slotted in and out as data becomes available. Previous work has shown phase 0 microdose studies demonstrating comparative PK data between IV microdose (100µg) administration with oral/ IV therapeutic dose for medications including clarithromycin and paracetamol [4, 5].

Phase 0 studies have the potential to improve pre-clinical candidate selection by providing human *in vivo* data earlier in the development process than with traditional approaches [6, 7]. A sub-therapeutic dose (100μg or 1/100^th^ of the anticipated minimal effective dose, whichever is smaller) of a novel therapeutic agent is administered to human participants. Participants can be healthy volunteers, or patients with the disease of interest, thereby providing relevant clinical data at the beginning of the drug development pipeline. Future work can formalise the criteria required to decide which candidate molecules should progress from phase 0 to phase 1 trials; but currently the concept can help select a lead candidate. Previous microdosing studies have given the confidence to terminate compounds with unfavourable qualities [8, 9]

A novel concept within the scope of phase 0 trials is Intra-target Microdosing (ITM) studies. With this approach, the microdose is administered in a controlled manner to a particular body compartment to allow for therapeutic drug levels at a local level but well within the sub-therapeutic systemic dose [10]. The concentrations of compound can be calculated to ensure that the target tissue or organ is exposed to a minimal effective dose, and so would not exceed that of a usual phase 1 study. If a compound is anticipated to have cytotoxic qualities, identified through preclinical data or rodent toxicology studies, then escalating titrations can be utilised. Highly sensitive methods of analysis including mass spectroscopy and proteo/lipidomics allow for ITM studies to provide data on mechanism of action and cell target engagement. Burt et al present an example ITM study of insulin within a tourniquet-ed hand [11]- utilised as a model example as the hand represents approximately one hundredth of the body volume in total.

There is a worldwide network of research groups who have published their work utilising a phase 0/ ITM method [12]. The Jonas lab at Harvard Medical School have used bio-micro devices alongside optical modalities to study drug effects at the micro-environment level [13, 14]. Presage Biosciences led by Dr Klinghoffer have used their Comparation in Vivo Oncology (CIVO) platform to evaluate drugs in fluorescently labelled and traceable ‘columns’ injected into tumours, with completed clinical trials in head and neck tumours. Beaton et al have published their phase 0 VEROnA study utilising vandetanib-eluting radio-opaque embolic compounds injected trans-arterially prior to hepatocellular carcinoma resection – a similar method to clinically established transcatheter arterial chemoembolization of liver tumours. Safety and tolerability of the procedure were demonstrated, as well as concentrations of vandetanib in the resected tumour [15]. Sjögren et al have utilised a microdose method as part of their preclinical studies on novel analgesic agents [16]. The Pandemic Science Hub at the University of Edinburgh have delivered pulmonary microdosing studies spanning matrix metalloprotease (MMP) inhibitors and fluorescent compounds [63–66] and have an active ITM study- ‘Micro’. This is a bronchoscopic delivered, first-in-human intra-pulmonary ITM study assessing an inflammasome inhibitor in interstitial lung disease and bronchiectasis.

An ITM style approach utilising microcassette drug delivery for lung cancers has been performed in humans [13]. The microcassette method allows for multiple compounds to be loaded to the device and administered simultaneously to human subjects. More general, non-pulmonary applications have shown its potential for PK assessment utilising LC-MS/MS methods, as well as drug-drug interactions at a tumour micro-environment [13, 14, 17, 18].

## Limitations of current drug development pathways

The process of drug development from the initial novel compound stage is complex, expensive, and ineffective, although in recent years there has been an increase in Food and Drug Administration (FDA) approved compounds. From 2012 -2022 the numbers of small molecule drugs approved by the FDA was 266. The median number of new molecular entities (NMEs) approved per year is 25, with a high of 37 in 2018 and a low of 9 in 2016 [19]. The process is increasingly expensive despite company mergers, reorganisation and other attempts to improve efficiency [1, 20]. Traditional drug development research and development (R&D) costs are high and have increased substantially over time [21].

The traditional model of drug development has 3 phases of clinical trials:

- Preclinical data: studies performed using cell lines, organoids, organ on a chip and animal testing.
- Phase I: Generally, dose escalation trials performed on a small number of healthy volunteers. Safety is the main outcome.
- Phase II: Small trials where the drug is administered to patients with the disease. Effectiveness, drug dosage and safety are the usual outcomes. Phase Ib/IIa trials can be performed in tandem where dose escalation is performed to determine the maximum tolerated dose, as well as the recommended clinical dose.
- Phase III: Larger trials where the drug is administered to patients with the disease. The drug is tested at the therapeutic dose, outcomes are effectiveness and safety. The gold standard phase III trials are large, randomised placebo-controlled trials or testing the drug against standard treatment.

Phase 0 trials sit between the preclinical stage and phase I trials.

Analysis has shown that between 2006 and 2015 the likelihood of approval from phase I for all compounds was 9.6% and 11.9% for indications outside of oncology [22]. The majority of drugs dropped out at the phase II level, with only 30.7% advancing to phase III trials. For compounds where the disease in question is a respiratory condition, the likelihood of approval from entry into a phase I trial was 12.8%. Again, phase II trials saw the greatest attrition rate with the likelihood of approval for a respiratory compound 19.6% from entry into a phase II trial. There was noted to be a high variability of successful approval rates between disease areas, with haematology having the highest. NME drugs have a 3 times lower success rate of approval when compared to drugs with already approved classes and targets. Phase II is typically seen as having a high rate of attrition as compounds generally move from patient safety and toxicology testing in phase I without having robust data showing clinical effectiveness or target engagement within humans. This is in part since phase I typically study healthy volunteers and so is an imperfect model to study therapeutic effects of drugs. Animal models too can be poor predictors of human drug efficacy and thus the overall effectiveness of the process is reduced [23–26]. Drug development in respiratory medicine is hindered by various factors including poor animal models, our incomplete understanding of underlying pathology, as well as the challenges of developing drugs for inhalation delivery [27]. The authors feel that there is the potential for bronchoscopically delivered ITM studies to provide early mechanistic data with pharmacologically relevant localised compound delivery.

### Phase 0/ microdose trials

Phase 0 clinical trials can establish at the very earliest opportunity whether a drug is modulating its target, and consequently whether further clinical development is warranted. The basic principle behind phase 0/ microdose trials is that a miniscule amount of the compound under investigation is administered to the human subject. Dose is limited to 100µg or less than 1/100 of the No Observed Adverse Effect Level (NOAEL) or the pharmacologically active dose and so patient safety is not put at risk, despite the early stage of development. Phase 0 trials can provide very early readouts on PK, PD and target engagement, thereby reducing development timelines by demonstrating proof of concept data earlier in the development pipeline [7, 28, 29]. This approach may allow investigators to recruit typically overlooked and understudied groups, despite the trials occurring at a first-in-human stage – for example pregnant women, children, and frail patients with more extensive disease, all examples of patient groups that are typically excluded from early-phase clinical development and often from drug development entirely (e.g., pregnant women) [7].

The International Conference on Harmonisation M3 Guidelines provide a regulatory framework for governing phase 0 trials with a spectrum of 5 approaches ranging from single to multiple exposures [29]. The 5 approaches of phase 0 trials are summarised by Burt et al and laid out in Table 1 below [6].

**Table 1:** Summarising the 5 approaches of phase 0 trials. NOAEL: No Observed Adverse Effect Level. AUC: Area Under Curve. GLP: Good Laboratory Practice

	**Number/ duration of doses**	**Maximum dose**	**Preclinical Requirements**
Approach 1	1	100µg and 1/100^th^ of NOAEL	Extended single dose toxicity in rodent; GLP
Approach 2	5 (6 half-lives between doses)	Each dose; 100µg and 1/100^th^ of NOAEL	7-day repeated dose toxicity in rodent; GLP
Approach 3	1	Starting at subtherapeutic dose and moving into the anticipated therapeutic range but < ½ NOAEL	Extended single dose toxicity in rodent and non- rodent; GLP
Approach 4	Multiple <14 days	Starting dose: <1/50 NOAEL; Into the anticipated therapeutic range but 10^th^ preclinical AUC if no toxicity, or <NOAEL	14-day repeated dose toxicity in rodent and non-rodent; GLP
Approach 5	Multiple <14 days	Starting dose: <1/50 NOAEL; Into the anticipated therapeutic range but< non‐rodent NOAEL AUC, or <½ rodent NOAEL AUC	14-day repeated dose toxicity in rodent and non-rodent; GLP

### Phase 0/ microdose vs traditional Phase I First in Human trials

In contrast to a Phase 0 trial, there are far more rigorous pre-clinical and regulatory requirements to meet before a traditional Phase I first in human (FIH) can be initiated. There are numerous guidance documents for developing preclinical safety programs to support a FIH trial [30–33] and, in general, nonclinical testing strategy is based on global and regional guidelines. The guidelines are then tailored to drug and target mechanistic predictions, specific drug characteristics and the proposed FIH clinical testing plan.

The global drug development community has long recognised the need to expedite drug discovery programmes and regulators in various countries have now enabled Phase 0 microdosing studies to take place under more relaxed regulatory requirements. Given i) Phase 0 studies have no therapeutic intent, ii) the doses and drug exposures are much lower and iii) significant drug-related adverse events are not anticipated, regulators allow more limited (single-dose or short-course) preclinical toxicology studies to be used to establish margin of safety rather than dose-limiting toxicities. Furthermore, because Phase 0 studies only require a small amount of study drug, full-scale, clinical Good Manufacturing Practice (GMP)-grade commercial manufacturing is not required before trial initiation. Good Laboratory Practice (GLP) product will often suffice [34]. Phase 0 trials can therefore be initiated earlier than traditional phase I studies, are subject to less demanding and costly pre-clinical and regulatory requirements, thus enabling them to measure drug target effects in humans much earlier in the clinical development pathway.

Due to the small doses of drugs involved, highly sensitive methods of analysis are utilised in the phase 0 approach. Most studies utilise LC-MS/MS, PET and accelerator mass spectrometry (AMS) [6, 35]. PET studies allow for measuring the pharmacokinetics of drugs labelled with positron-emitting radionuclides in different tissues and organs of the body. LC-MS/MS is generally cheap and readily available and can provide drug concentration data on various bodily fluids including plasma, urine and bronchoalveolar lavage fluid (BALF). Plasma is the most frequently tested bodily fluid for PK data.

Phase 0 trials typically utilise these sensitive methods and extrapolate data to larger, clinical and therapeutic doses. Studies have now displayed the ability to extrapolate data from microdosing trials to calculate the PK of a drug despite the small doses utilised [4, 36–38]. This is usually performed by performing noncompartmental analysis and determining the fold difference between the dose normalised PK data between the microdose and full dose [4, 39, 40]. A two-fold acceptance criterion is used. Other models have used a naive pooled data modelling approach [36]. Phase 0 approaches have been validated on both established medications [39], as well as on compounds at early phase of development [41]. It must be acknowledged that some compounds have not been shown to have predictable extrapolation using this approach [42]. A review by van Nuland et al which studied 46 compounds tested in phase 0 trials found that there was acceptable predictability for 68% of compounds administered orally and 94% of compounds administered intravenously [5]. They commented that nonlinearity is likely due to saturation of the enzyme or transporter systems but felt that their review supported the use of phase 0 clinical trials for gathering early PK data. Bosgra et al present a decision tree that can be utilised to predict if a compound is likely to display nonlinear properties and less likely to be suitable for an early phase 0 approach [37].They validated their method comparing 10 published compounds where phase 0 and therapeutic dose PKs have been calculated. Their paper outlines predictors of nonlinear PK; gastrointestinal dissolution, intestinal and hepatic efflux transport, metabolism & uptake transport, plasma protein binding and active renal elimination. Appropriate selection of early compounds, complementing other preclinical PK data, can reduce resource waste within drug development.

Phase 0 trials have been performed on various drugs with respiratory disease indications, particularly chemotherapy agents [38, 43–46]. Wang et al performed a pilot microdosing clinical trial with carboplatin, a platinum-based chemotherapy agent used frequently with non-small cell lung cancer (Clinical Trials.net identifier NCT01261299). 1% of the therapeutic dose of radio labelled carboplatin was administered via IV infusion and plasma PK data was quantified via an AMS method. The calculated PK was consistent with that of therapeutic doses. Van der Veldt et al have utilised the phase 0 methodology in the study of docetaxel in lung cancer patients. PET-CT imaging was used to show drug uptake of microdose of carbon-11 labelled docetaxel. 34 patients with lung cancer were recruited to receive [^11^C]docetaxel. As well as the effect of tumour perfusion and size, the team were able to show that premedication with dexamethasone had a negative effect on tumour uptake of the radiolabelled drug [47]. They have previously published work validating the method with comparison to microdose and therapeutic dose infusions [48].

Ordonez et al have published work with carbon-11 labelled rifampin in the study of rifampin sensitive pulmonary tuberculosis (TB) infection. It is vital to achieve appropriate antimicrobial concentrations to treat complex infections such as TB, and plasma concentrations are often a poor surrogate for infections site levels, especially cavitatory disease. In their study, 12 patients with TB were given an intravenous microdose of [^11^C]rifampin and dynamic PET-CT was used to evaluate drug concentrations in compartments and organs. [^11^C]Rifampin levels were low within pulmonary cavity walls, and levels were heterogenous in pathologically distinct TB lesions even for separate lesions in individual patients [49]. They describe the potential for treatment duration shortening based on intralesional drug levels.

It is also possible to utilise a microdose approach to understand more localised lung PK. Lappin et al have published the results of a phase 0 trial of AR-709, a novel diaminopyrimidine antibiotic [8]. Four healthy patients received 2 doses, 7 days apart, of 100µg of radio labelled AR-709, intravenously initially and then orally. Drug concentration data was analysed via LC-MS & AMS from peripheral plasma. Following this, 15 healthy individuals received 100µg of AR-709 intravenously and 8-12 hours later underwent bronchoscopy and lavage. Plasma, BALF, alveolar macrophages and bronchial mucosal samples were analysed via AMS. They were able to show that up to 12 hours after IV dosing the concentration of AR-709 was up to 15 times higher in bronchial mucosa and up to 200 times higher in alveolar macrophages. Whilst these results were promising they also found that the oral bioavailability was poor and so AR-709 was not a suitable candidate for further development.

## Regulation and toxicology requirements

### Phase 0 regulatory framework

Phase 0 clinical studies have been performed for over 20 years [35, 50, 51]. In 2009, microdosing frameworks in the USA, Europe and Japan were integrated, harmonised and ratified under the International Conference on Harmonization (ICH) M3 “Guidance on Nonclinical Safety Studies for the Conduct of Human Clinical Trials and Marketing Authorization for Pharmaceuticals (ICH, 2009) [52]. Five phase 0 approaches are described in Section 7 of the guidelines and cover first-in-human testing in patients, simultaneous testing of multiple drug candidates (cassette microdosing), ITM and intravenous administration of drugs. The phase 0 regulatory approach across the main jurisdictions is directly proportional to the risks associated with drug exposure. Exposure to the drug is at the lowest end of therapeutic-level exposure range, and with regards to ITM, no more than 1/100^th^ of the body mass exposed to the drug. The therapeutic-level exposure at the target is typically brief (in the region of seconds) before the drug is cleared from the tissue.

The regulatory framework intends to encourage flexibility and challenge drug development timelines by reducing pre-clinical and manufacturing requirements, thereby bringing forwards candidate prioritisation and de-prioritisation decisions. It could be argued that Sponsors and investigators are not making full use of the flexibility that phase 0 offers, and this could be partially driven by a lack of understanding of the manufacturing and toxicology requirements and how the clinical data can support further drug development.

### Phase 0 toxicology requirements

Phase 0 provides early data about the how drugs behave in humans, which can in turn improve how we model drug toxicity and efficacy prior to embarking on larger clinical studies. If we can screen and model the profiles of drugs in the human before we reach phase I, fewer animal studies are required. Phase 0 thereby directly supports the UK and European legislation that requires that Replacement, Reduction and Refinement of animal procedures (the Three Rs) are implemented wherever possible.

Given doses and exposures are low in phase 0 studies, drug-related adverse events are not anticipated, and therefore more limited (single-dose or short-course) preclinical toxicology studies to establish margin of safety can be undertaken. In general, preclinical toxicology studies conducted to support a phase I study should demonstrate that a dose 100 times greater than the proposed clinical dose would not cause harm to the patient or healthy volunteer. Toxicology requirements are outlined in the ICH M3 guidance for each of the five example phase 0 approaches. The exposure generally corresponds with the extent of the preclinical studies that need to be undertaken before human testing. For example, a single microdose, requires only one, extended single dose toxicity study in one animal species, typically rodents, with follow-up at day 14. However, microdosing that elicits concentrations in the low therapeutic dose range at the target site (<14 days), with no therapeutic intent, and not intended to evaluate tolerance, requires a 2-week repeated dose toxicity study in rodents followed by a confirmatory study in a non-rodent species for the duration of the intended exposure in the phase 0 study. The main take home message regarding the toxicology requirements in phase 0 studies is that they can be specifically tailored to the needs of the drug, planned microdosing approach and proposed clinical study and can be discussed with the regulators.

### Intra-Target Microdosing (ITM)

Intra-Target Microdosing (ITM) studies are a novel approach to phase 0 trials where a microdose (again limited to 100µg or 1/100 of the NOAEL) is administered to a closed compartment within the body so that local therapeutic-level dose is achieved. The overall systemic dose remains tiny and well below a therapeutic level, and if administered to approximately 1/100^th^ of the body, it is possible to study therapeutic PDs and mechanism of action at the local level. Compounds may be administered via the IV route, or for example intra- dermal or intra-muscular [16]. Proof of concept have been performed on rodent models [53] and in feasibility studies where a microdose of insulin has been administered into a hand with a tourniquet applied [11]. Published ITM studies in the lung have thus far focused on the potential to study chemotherapy agents at the tumour microenvironment level.

### ITM and lung cancer

The tumour microenvironment contains a complex, heterogenous ecosystem where intrinsic immune alterations can shape not just tumour growth and progression but also response to anti-tumour therapy. The is true for lung cancer, and recent advances in immunotherapy show the potential in what was previously a disease with poor prognostic outlooks. ITM studies allows for the study of cancer drug therapy within the tumour microenvironment itself- a so called ‘lab-in-a-tumour’ approach. In addition, it is possible to study ‘drug-drug’ interplay, as well as individualised patient disease response to drugs.

Jonas et al have developed a Multiplex Implantable Microdevice Assay (MIMA) microdevice to study multiple anti-cancer therapies in combination [54]. The MIMA device is a 5mm long biocompatible chamber which is implanted onto the surface of the tumour *in vivo*. Nanodoses (defined by the authors as doses limited to nanolitres) of drugs can be loaded in the 18- well device alone, or in combination, and will be released to the tumour via passive diffusion. Tumours are harvested following a period of drug delivery and drug response measured. Their initial work was performed on murine models of breast cancer [14]. The MIMA devices were loaded with 7 anti-cancer agents including doxorubicin and paclitaxel and implanted into the living mouse tumour. The tumours were harvested after 3 days, and tissue sections were cut in a way as to study the local effects of the various agents. Spatial single cell analysis is utilised to study the cellular phenotypes and immune response to the chemotherapy agents at a microenvironment level.

The team have published a method of implanting and retrieving a drug delivery microdevice into murine tumours using ultrasound and CT guidance [55]. The devices were left *in situ* within the living mice for 24 hours prior to retrieval and local tumour tissue was removed alongside the device for study of drug effect. Retrieval is similar to a needle biopsy of the tumour with a custom, 14G bi-axial needle. The team suggest that this method can be used for both cancer and non-cancer drug study in a broad range of clinical areas and organs. Potential future development includes the ability of the device to transmit electronic and optical data.

In addition, their team has developed an implantable microdevice that alongside drug microdoses, delivers microdoses of a fluorescent cell death assay [54]. This assay accumulates at sites of increased cell death induced by the chemotherapy agent. The microdevice itself is interfaced with a fluorescent microscope and the imaging capabilities can visualise individual cells as small as 10µm in diameter up to a distance of 300µm from the probe. Thus, this microdevice can image the effects, in real time, of microdoses of chemotherapy agents delivered to tumours. These devices have been published on murine models but there are plans to utilise them in human trials.

Tsai et al have presented the results of their fist in human clinical trial of an intrathoracic implantation of a multidrug eluting microdevice [13]. Their device – named NanoNail^TM^ was placed intraoperatively in the tumours of 5 patients with non-small cell lung cancer in order to measure *in situ* drug sensitivity to 12 chemotherapy agents. The NanoNail^TM^ was removed alongside the tumour and the area adjacent to the device- where the drugs had been eluted – was analysed for markers of apoptosis and cell proliferation. This study demonstrates the ability to perform ITM studies utilising microcassettes and multiple drug effects, which can be incorporated during thoracic surgery and lung resection.

A pilot study has been registered (Clinical Trials. Net identifier NCT03972228) which plans to recruit 5 patients with surgically resectable lung cancer [56]. A microdevice loaded with microdoses of 19 chemotherapy agents will be implanted into the tumours at the time of resection and will be retrieved from the sample following retrieval. The study aims to show feasibility of implantation and retrieval, as well as early data on tissue response to the drugs.

Klinghoffer et al describe a similar microcassette device which can be used to assess up to 8 drugs in combination within a solid tumour [57]. Their model is designed for the study of superficial tumours and they have registered a phase 0 master protocol for the intra-tumoural microdosing of anticancer agents (Clinical Trials. Net identifier NCT04541108). 15 patients are to be recruited and each tumour will be injected with a CIVO device loaded with Pembrolizumab and a combination of novel chemotherapy agents. Following excision tumours will be assessed for quantification of cell death and immune cell biomarkers.

## Limitations

Not all agents are appropriate for phase 0/ ITM trials; as mentioned previously it is best where there is predictable and dose dependent PK/PD [58]. Extrapolation of PK remains a challenge for phase 0 trails but can be overcome with appropriate modelling [7]. Immunotherapeutic agents and checkpoint inhibitors in particular have the additional challenge of the complexity of the human immune system and the anti-cancer effect that may be distant to what is detected with ITM studies [59]. Previous ITM studies have shown promise in detecting immunomodulatory effects [14]. Heterogenicity within solid tumours presents the risk that any implanted device or injected compound may be an area not representative of the overall tumour, thus affecting any gathered data. Tsai et al’s paper presents an excellent overview of the learning process in placing a microdevice within a solid tumour during surgical excision [13]. For non-cancer phase 0/ ITM studies where the compound is delivered endobronchially appropriate pre-procedure radiology should direct administration.

The ethical implications of performing phase 0 trials have been explored. Participants cannot expect to have any chance of potential benefit, even accepting that there is only a tiny risk of local toxicity [60]. This is similar to early phase 1 trials where treatment benefit is not expected, however it is noted that here participants are often healthy volunteers. Hill discusses the ethics of phase 0 trials as it relates to a means-ends relationship, and if it is possible to replace the potential of therapeutic benefit with indirect benefit and benefit to others [61].

## Future ITM studies and the lung

**Fig. 1:**
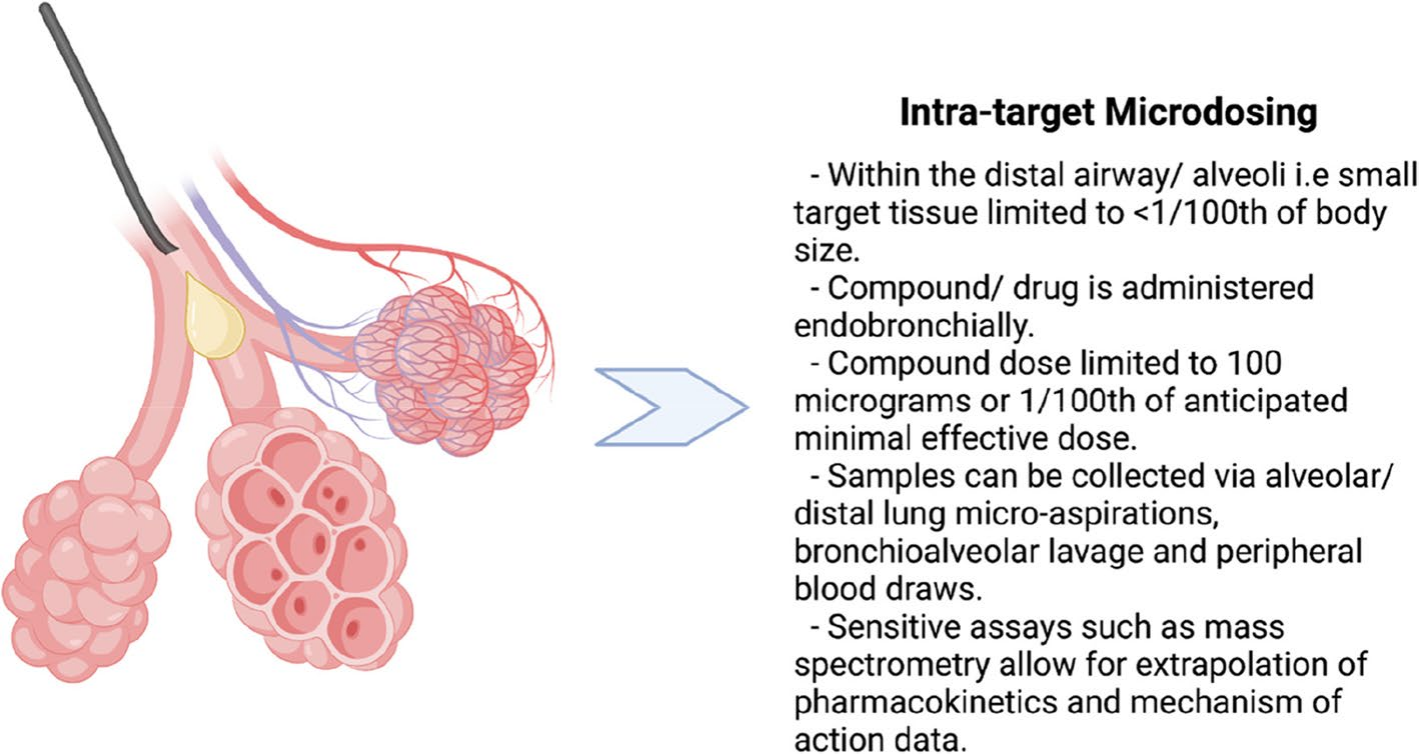
Schematic explaining the process of intra target microdosing within the distal lung

The Pandemic Science Hub has an active phase 0/ ITM study of bronchoscopic administration of a novel inflammasome inhibitor to the distal airway/ alveolar space of patients with interstitial lung disease and bronchiectasis. This study seeks to test the hypotheses that the compound, when administered endobronchially can attenuate alveolar macrophage inflammasome activity. Figure 1 contains a cartoon schematic demonstrating how the agent will be administered to the distal airways in an ITM fashion. 100µg will be administered to a contained space so that a therapeutic concentration is reached at a local level. Readouts will include IL-1ß as a marker of inflammasome activity, proteomics and LC-MS drug concentrations. We have utilised a human ex vivo lung model to plan and de-risk our approach for this trial- a method that has previously been described for drug development [62]. Our research group has experience in delivering small volumes of Smartprobe imaging agents at microdose levels in the distal lung using a bronchoscopic technique - these studies were not formal ITM, but rather alveolar endomicroscopy imaging studies utilising fluorescent probes [63–65].

## Summary

As we plan for a more sustainable future it is vital we improve efficiency in healthcare- both in how we deliver it and in the development of new treatments and drugs. Attrition of new compounds as they pass through the stages of clinical trials is a major drag on efficiency. Most drugs fail at phase II as they are found to be safe but have poor PK or mechanism of action. The use of phase 0 trials allows us to select the most promising pre-clinical compounds and demonstrate favourable PK/PD prior at the earliest stage of human testing. Specifically for lung disease, when a bronchoscopic approach is utilised, we can study topical application of drugs that may ultimately be administered via inhaler or nebuliser- avoiding the greater potential of side effects with systemic administration.

## Data Availability

No datasets were generated or analysed during the current study.
